# TMEM106C, BSG, COPE, CDCA8, KPNA2, LIG1, UQCRH, and CCT5: Predictive of Survival and Immunotherapy Resistance in Hepatocellular Carcinoma

**DOI:** 10.1155/humu/1465989

**Published:** 2026-02-10

**Authors:** Kai Yu, Minqi Chen, Wanchao Hou, Jinhua Lu, Qianhan Liu, Wanrong Zeng, Zhengcai Du, Xiaotao Hou, Erwei Hao, Jiagang Deng

**Affiliations:** ^1^ Guangxi Key Laboratory of Efficacy Study on Chinese Materia Medica, Guangxi University of Chinese Medicine, Nanning, China, gxtcm.com; ^2^ Guangxi Key Laboratory of TCM Formulas Theory and Transformation for Damp Diseases, Guangxi University of Chinese Medicine, Nanning, China, gxtcm.com; ^3^ Guangxi Collaborative Innovation Center of Study on Functional Ingredients of Agricultural Residues, Guangxi University of Chinese Medicine, Nanning, China, gxtcm.com; ^4^ University Engineering Research Center of Reutilization of Traditional Chinese Medicine Resources, Nanning, China; ^5^ College of Pharmacy, Guangxi University of Chinese Medicine, Nanning, China, gxtcm.com

**Keywords:** cellular senescence, gene signature, hepatocellular carcinoma, prognosis, single-cell RNA sequencing

## Abstract

**Introduction:**

Hepatocellular carcinoma (HCC) remains a leading cause of cancer‐related mortality worldwide, with cellular senescence playing a context‐dependent role in tumor progression and the immunosuppressive microenvironment. This study is aimed at identifying senescence‐related gene signatures through integrated single‐cell and transcriptomic analyses to construct a robust prognostic model for predicting survival and immunotherapy response in HCC patients.

**Methods:**

We obtained single‐cell RNA sequencing (scRNA‐seq) data from the Gene Expression Omnibus (GEO) database and transcriptomic data from The Cancer Genome Atlas (TCGA). The scRNA‐seq data were processed using the Seurat and Harmony packages for cell clustering and batch correction. Senescence scores were calculated via the AUCell package, and differentially expressed genes were identified using the limma package. Prognostic genes were selected through univariate and LASSO Cox regression (glmnet package) to construct a risk model, which was validated in multiple independent cohorts. Immune infiltration was assessed with single‐sample gene set enrichment analysis (ssGSEA), TIMER, and MCPCounter algorithms, and response to immune checkpoint blockade was predicted using the tumor immune dysfunction and exclusion (TIDE) platform. Experimental validation included qRT‐PCR, Cell Counting Kit‐8 (CCK‐8), wound healing, and Transwell assays in HCC cell lines.

**Results:**

A total of 80,997 identified cells were allocated to eight clusters, with an evidently higher percentage of natural killer (NK) cells in HCC samples. A higher senescence score was also seen in HCC samples, and poor prognosis was noticed in the patients of high senescence score group. Further, the DEGs were intersected with the genes highly expressed in Population 4 of NK cells to reveal their enrichment in cell cycle and cell division. Further, eight genes (*TMEM106C*, *BSG*, *COPE*, *CDCA8*, *KPNA2*, *LIG1*, *UQCRH*, and *CCT5*) with differential expression in HCC were applied to construct the risk model, which could stratify HCC patients into different risks and predict the prognosis. Besides, the high immune infiltration and expression levels of immune checkpoint–relevant genes yet poor immunotherapy response were noticed in HCC patients of high risk. Further validation tests have suggested that the knockdown of *CDCA8* repressed the malignant phenotypes of HCC cells.

**Discussion:**

This integrated analysis establishes a senescence‐related gene signature as a robust tool for prognostic stratification and immunotherapy response prediction in HCC. The model highlights the complex interplay between cellular senescence and the immunosuppressive tumor microenvironment, offering insights for personalized treatment strategies. Furthermore, the identified biomarker *CDCA8* represents a promising therapeutic target warranting further investigation.

**Conclusion:**

These discoveries provide novel evidence on senescence in HCC, which may tailor the pharmacological interventions to improve the clinical management.

## 1. Introduction

As the primary malignancy of hepatocytes, hepatocellular carcinoma (HCC) ranks sixth in the most prevalent malignancy and the fourth major cause of cancer‐associated mortality across the globe [1, 2]. Liver cirrhosis and chronic hepatitis, alcohol consumption, and some metabolic dysfunction, chronic infection of hepatitis B virus or hepatitis C virus are the main risk factors for HCC [3, 4]. Over the past decades, there has been significant advance made in the treatment of HCC, which has offered the chance of long‐term response, including surgery, orthotopic liver transplantation, and some ablative techniques like thermal ablation [5, 6]. Despite these modalities, a great number of patients are not eligible, thus making it of paramount importance to identify some new strategy to improve early HCC detection and prediction of therapeutic responses and survival for HCC patients [7].

It has been documented that cellular senescence in the liver could trigger the growth arrest in cells and has mostly been related to the repressed growth and progression in HCC [4]. Senescent cells are normally metabolically active and could secrete cytokines termed “senescence‐associated secretory phenotype” (SASP) factors. Therefore, cellular senescence can repress the progression of HCC via enhancing the clearance of hepatocytes through an orchestrated action of innate and adaptive immunity [8]. Senescent cells engage in intercellular crosstalk via dynamic changes in antigens presented by the major histocompatibility complex and/or surface proteins [9]. Senescent cells create either an anti‐inflammatory or pro‐inflammatory environment that dynamically influences tissue homeostasis and HCC development through such intricate intercellular communication [10, 11]. The identification of senescence‐related genes (SRGs) with potential diagnostic or prognostic efficacy in HCC has therefore become the objective of this study.

In the meantime, a study has been carried out to recognize transcriptome‐wide changes that contribute to malignant transformation of different tissue [12]. Increasing evidences have been also applying genetic prognostic models based on the Cancer Genome Atlas (TCGA) database to a wide range of human malignancies like HCC [13]. Accumulating studies have already addressed the senescence signatures in HCC with the application of both bulk RNA‐seq and scRNA‐seq, which have successfully pinpointed the HCC‐specific senescence pathways [14]. Here, in this study, we further characterized the SRGs in HCC by applying the data from publicly available databases and constructed a relevant prognostic model for immunotherapy response and survival prediction in HCC, with the hope to improve the efficacy of clinical management on HCC.

## 2. Methods

### 2.1. Data Source

The following data were collected for our analyses:

1. The scRNA‐seq data of HCC containing three HCC and three healthy liver samples were downloaded from the dataset GSE162616 [15].
2. TCGA was accessed to download the data of clinical information and gene expression of patients with liver hepatocellular carcinoma (LIHC). The FPKM value in the RNA‐seq data was transformed to transcripts with TPM and the log_2_ transformation was carried out. Samples with survival > 30 days were retained, and 342 HCC samples and 50 control samples were accordingly collected.
3. The clinical information of HCC patients and the gene expression data were obtained from the dataset GSE43619 (platform: GPL10558). A total of 88 tumor samples were included [16].
4. The ICGC‐LIRI‐JP cohort (http://lifeome.net/database/hccdb/home.html) was downloaded from the HCCDB database [17]. A total of 211 HCC and 177 control samples were incorporated.

### 2.2. Data Processing

The “Read10X” function of the “Seurat” R package was applied to read the scRNA‐seq data of each sample [18, 19]. Cells with gene count between 200 and 3000 and with mitochondrial gene count < 10*%* were retained and standardized using the “SCTransform” function. Following the dimensionality reduction via principal component analysis (PCA), the intersample batch effects were removed using the “Harmony” R package [20]. The “RunUMAP” function was applied for dimensionality reduction, and the “FindNeighbors” and “FindClusters” functions (parameters: dims = 1 : 25 and resolution = 0.1) were employed for clustering the cells. The obtained cell clusters were finally annotated using the markers in the CellMarker2.0 database.

Additionally, the “AUCell” R package was applied to calculate the senescence score in different cell populations [21].

### 2.3. DEGs and Functional Enrichment Analyses

The data from TCGA were applied for the analysis to reveal the DEGs with the help of the “limma” R package [22]. The relevant DEGs were sorted at the parameters |log2FC| > 1 and adjusted *p* value < 0.05. The effects of DEGs were explored according to the three terms of gene ontology (GO) enrichment analysis.

### 2.4. Construction and Validation of the Risk Model

The prognostically relevant genes were firstly sorted via univariate Cox regression analysis and reduced using LASSO Cox regression analysis by the “glmnet” R package [23]. The key genes and their coefficients were obtained via stepwise regression and the risk score for the model was calculated using the Formula (1).

(1) Risk score=Σ βi×Expi

(*β* in the formula is the coefficient number, and *i* represents the gene level of expression.)

The patients were then stratified into high‐ or low‐risk groups based on the median of the calculated risk score, and the Kaplan–Meier (KM) curve was plotted for survival prognostication. The relevant receiver operator characteristic (ROC) curve was plotted, and the AUC value was calculated based on the “timeROC” R package [24].

### 2.5. Immune Infiltration Analysis

The immune cell infiltration degree was estimated using two complementary algorithms, “MCPCounter” and “TIMER”, to ensure robust and comprehensive profiling of the tumor microenvironment. The scores of 28 types of tumor‐infiltrating lymphocytes (TILs) were further calculated using the “GSVA” R package [25], which enables a sensitive, nonparametric assessment of immune cell activity based on gene set variation analysis, thereby providing a detailed landscape of lymphocyte subsets within the samples.

### 2.6. Immunotherapy Response Prediction

The difference on the predicted immunotherapy response in patients of the two groups was compared using the “TIDE” algorithm. Meanwhile, the level of immune checkpoint–relevant genes in the two groups was also compared, and the corresponding results were visualized in a heatmap.

### 2.7. Cell Culture and Transfection

Human immortal adult liver epithelial cell line THLE‐2 (C5664, RRID: CVCL_3803) and HCC cell line HuH7 (C5176, RRID: CVCL_0336) were all purchased from BD Bio (Hangzhou, China) and cultured as follows: THLE‐2 cells were grown in bronchial epithelial cell growth medium (CC‐3170, Lonza, Basel, Switzerland) containing 10% fetal bovine serum (FBS, F814‐500, BD Bio, China), epidermal growth factor (5 ng/mL, 01‐107, Merck Sigma, Darmstadt, Germany), and Phosphoethanolamine (70 ng/mL, P0503, Merck Sigma, Germany). HCC cells HuH7 were grown in high‐glucose DMEM (L1004‐500, BD Bio, China) supplemented with 10% FBS. All cells were identified via short tandem repeat analysis and confirmed negative for mycoplasma contamination, which were incubated in an incubator at 37°C with 5% CO_2_.

Based on the results and the existing studies [26], *CDCA8* was applied as the target gene of interest for the knockdown assay. Accordingly, the small interfering RNA targeting *CDCA8* and the corresponding negative control with scramble target sequence were all customized and ordered from GenePharma (Shanghai, China), which were then transfected into HCC cells HuH7 using Lipofectamine 2000 transfection reagent (11668027, Invitrogen, Carlsbad, CA, United States) as per the manuals. The target sequence of the small interfering RNAs was listed in Table S1.

### 2.8. Quantitative Reverse Transcription Polymerase Chain Reaction (qRT‐PCR)

The TriZol reagent (15596‐026, Invitrogen, United States) was employed to isolate total cellular RNA as per the manuals and the concentration of isolated RNA was thereafter quantified in a spectrophotometer (ND‐2000, ThermoFisher Scientific, Waltham, MA, United States). A commercial 1st strand cDNA synthesis kit (D7170S, Beyotime, Shanghai, China) was applied to synthesize the cDNA from 1 *μ*g of total RNA, and the PCR was then performed using SYBR Green qPCR Mix assay kit (D7260, Beyotime, China) and CFX384 real‐time PCR detection system (Bio‐Rad, Hercules, CA, United States) with the indicated primers (detailed information is available in Table S2). The relative mRNA level was gauged with the 2^−*ΔΔ*ct^ method using *GAPDH* as internal control [27].

### 2.9. Cell Viability Assay

The transfected HCC cells HuH7 were seeded in a 96‐well plate at the concentration of 2 × 10^3^ cells per well and cultured for 24, 48, and 72 h, following which 10‐*μ*L CCK‐8 cell viability test solution from the assay kit (C0037, Beyotime, China) was added for an additional 4‐h culture. Thereafter, the OD value was read using a microplate reader (iMark, Bio‐Rad, United States) at 450 nm.

### 2.10. Cell Migration Assay

The migration of HCC cells HuH7 following the transfection was explored via scratch assay. Concretely, the transfected cells were grown in a 6‐well plate at the concentration of 1 × 10^5^ cells/well and cultured overnight. Thereafter, a 200‐*μ*L pipette tip was applied to make a scratch on the monolayer once cells were completely confluent, and cells were further incubated in an incubator at 37°C for 48 h. An inverted optical microscope (IM‐300, Optika Microscopes, Ponteranica, Italy) was adopted to observe the migrated cells and the degree of wound closure (%) was additionally evaluated.

### 2.11. Cell Invasion Assay

Prethawed matrix gel (C0372, Beyotime, China) was added to the top transwell chamber (pore: 8 *μ*m, code. 3422, Corning, Inc., Corning, NY, United States) beforehand and 2 × 10^4^ transfected HuH7 cells were populated in the chamber with 200‐*μ*L serum‐depleted media, whereas the bottom transwell chamber was filled with 750‐*μ*L complete culture media with 10% FBS. Following the culture period of 48 h, a cotton swab was applied to remove the cells in the upper chamber, and those cells invaded to the bottom chamber were serially fixed in 4% paraformaldehyde (P0099, Beyotime, China) for 10 min and stained in crystal violet (C0121, Beyotime, China) for another 10 min. All invaded cells were finally observed under an inverted optical microscope to quantify the number as needed.

### 2.12. Statistical Analyses

All computational analysis was realized using R software (Version 3.6.0), and all data of laboratory assays were processed via GraphPad Prism software (Version 8.0.2). The Wilcoxon test was applied to compare the difference in two continuous variables, and the log–rank test was adopted to compare the survival of patients in different groups. For the experimental data, unpaired *t*‐test, one‐way, and two‐way ANOVA were applied to compare the data. All data with statistical significance were denoted by the asterisks at the threshold of *p* < 0.05, and those without statistical significance were marked with “ns” (nonsignificant, *p* > 0.05).

## 3. Results

### 3.1. Identification of SRGs in HCC via scRNA‐seq

The data of scRNA‐seq were analyzed in the beginning to reveal some SRGs in HCC. Following the procedures of cell filtering, standardization, dimensionality reduction, and clustering, 80,997 cells were identified and allocated to eight main clusters with their distinct markers (Figure 1a,b). The calculation on the percentage of these eight cell clusters in HCC and healthy samples suggested that the percentage of NK cells was evidently higher in HCC samples (Figure 1c,d). Thereafter, all NK cells were extracted and reclustered into five main populations. The senescence score of these populations was additionally calculated, revealing a relatively higher score of Population 4 (Figure 1e,f). These findings revealed the specific cell populations for our research and paved the way for our additional analyses on the genes of interest in HCC.

Figure 1Identification of SRGs in HCC via scRNA‐seq. (a) UMAP plot on the single cell in HCC based on the dataset GSE162616 to reveal specific cell clusters. (b) The expressions of cell marker genes specific to each cell cluster in bubble plot. (c–d) Difference in the percentage of each cell cluster in the HCC and healthy samples. (e) UMAP plot on the reclustering result of NK cells. (f) The calculated senescence score of each population of NK cells.

**Figure:**
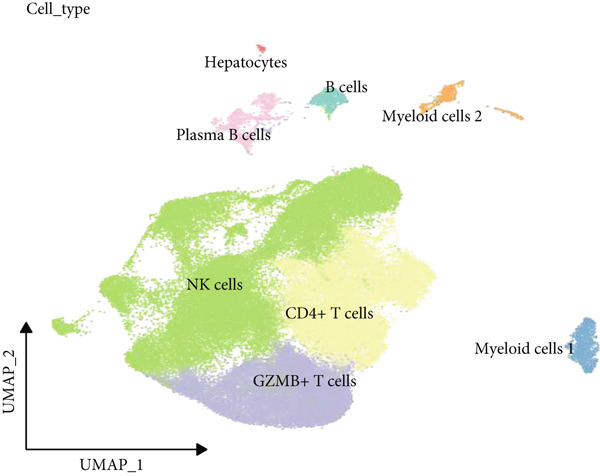
(a)

**Figure:**
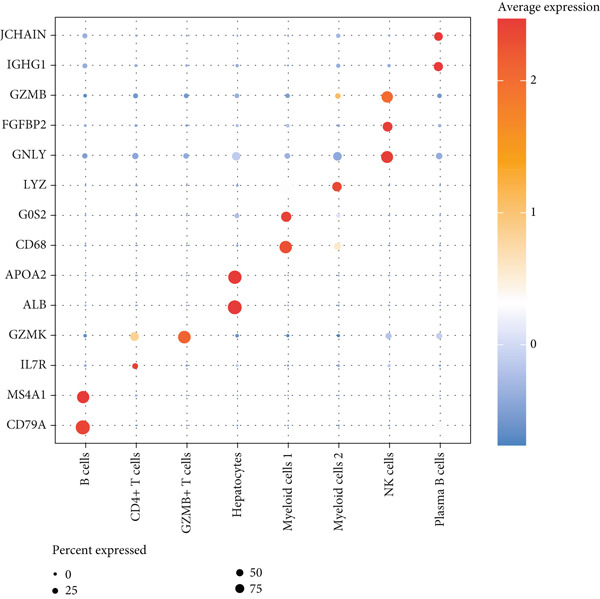
(b)

**Figure:**
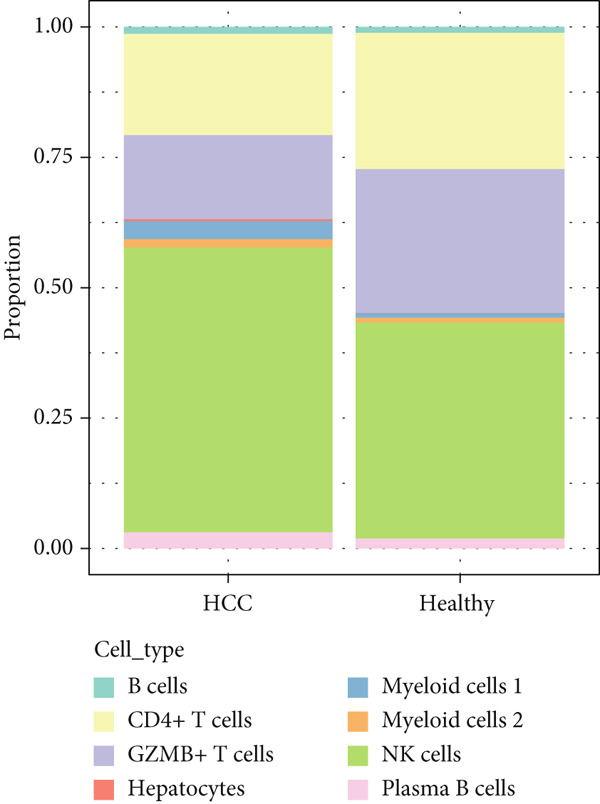
(c)

**Figure:**
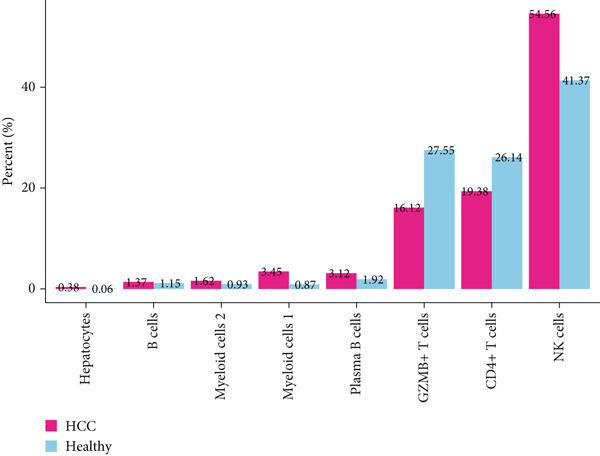
(d)

**Figure:**
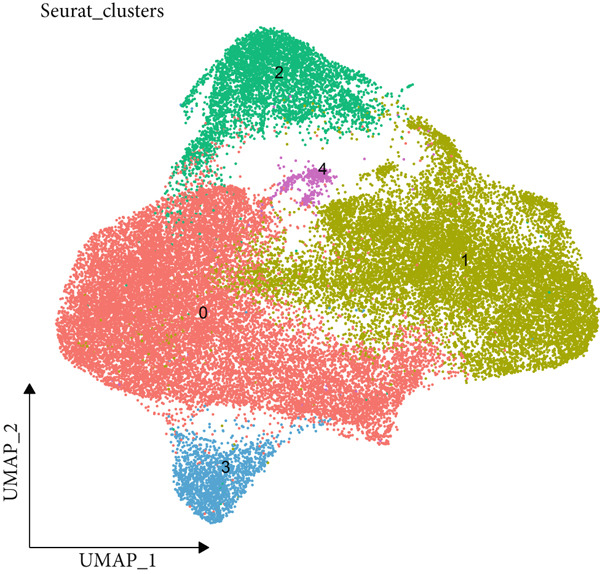
(e)

**Figure:**
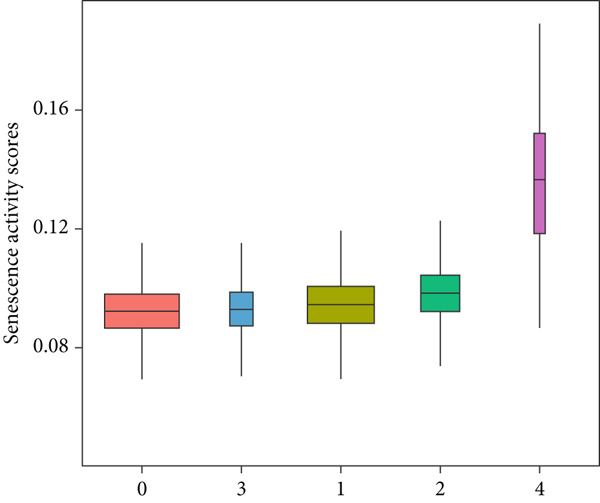
(f)

### 3.2. Relationship Between the Senescence Score and the Progression of HCC

The senescence score of samples in TCGA was calculated via ssGSEA, and a higher senescence score was seen in HCC samples than that in healthy samples (Figure 2a). Then, the HCC samples were allocated to the high or low score group by the median value of the senescence score, and the survival of patients in these two groups was compared. A poor prognosis was noticed in the high score group (Figure 2b), and the senescence score gradually increased with the severity of the clinical stages (Figure 2c,d). To validate our discoveries, similar analyses were also implemented on the ICGC‐LIHC‐JP cohorts, and similar results were seen as well. In other words, the senescence score was higher in HCC samples, and a higher senescence score was indicative of a worse prognosis (Figure 2e,f), thus demonstrating the involvement of senescence in HCC.

Figure 2Relationship between the senescence score and the progression of HCC. (a) Comparison on the senescence score in HCC and normal samples using the data from TCGA. (b) The association between the survival probability (%) and the senescence score using the data from TCGA. (c–d) The relationship between the clinical stages and the senescence score. (e) Comparison on the senescence score in HCC and normal samples based on the data from ICGC‐LIHC‐JP cohort. (f) The association between the survival probability (%) and the senescence score using the data from ICGC‐LIHC‐JP cohort. The data with statistical significance were denoted with asterisks ( ^∗∗∗∗^ *p* < 0.0001).

**Figure:**
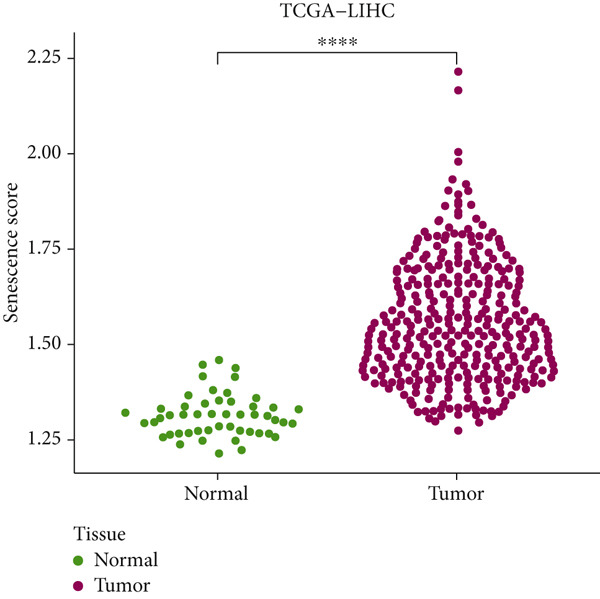
(a)

**Figure:**
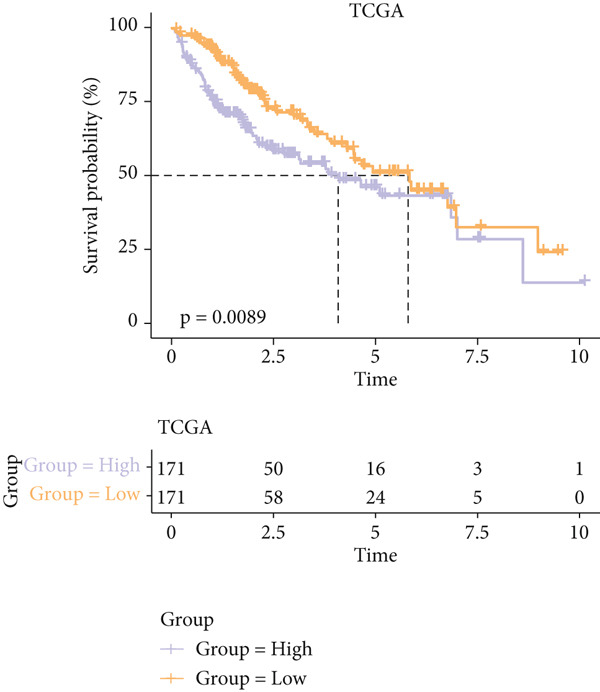
(b)

**Figure:**
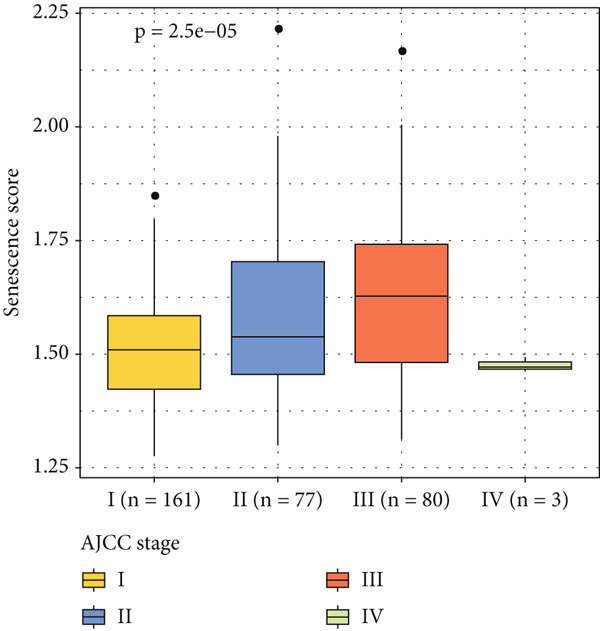
(c)

**Figure:**
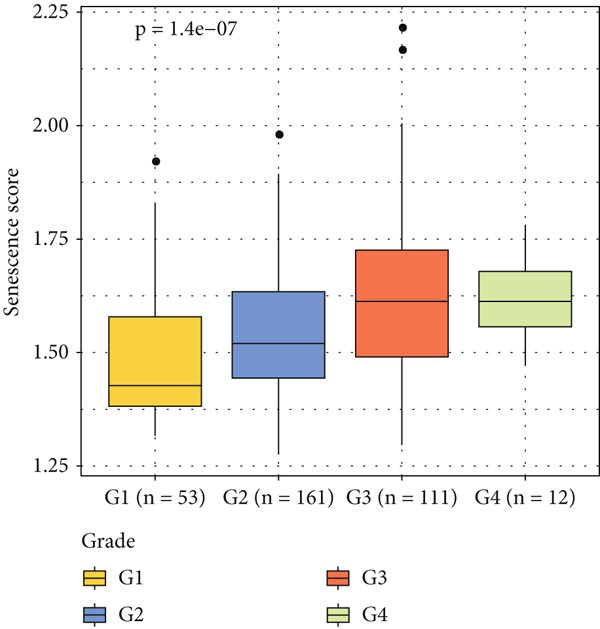
(d)

**Figure:**
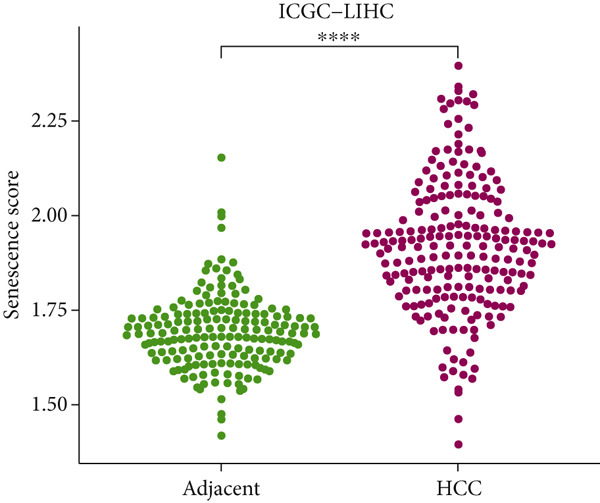
(e)

**Figure:**
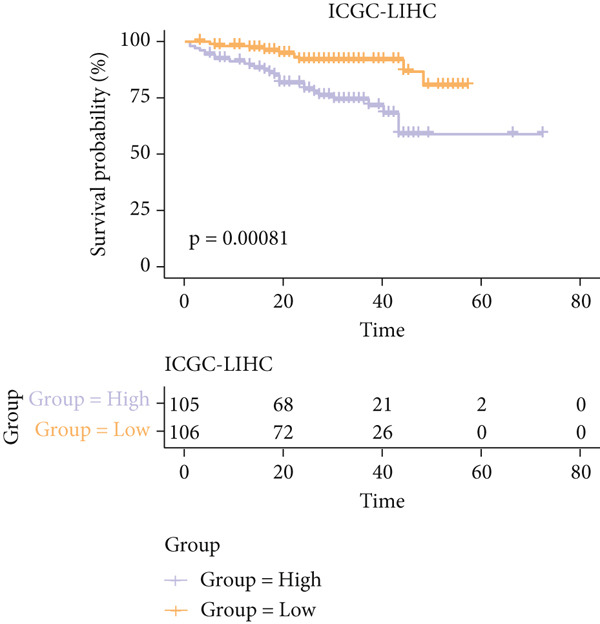
(f)

### 3.3. Sorting on the Feature Genes and Functional Enrichment Analysis

The DEGs of HCC and normal samples in TCGA were firstly analyzed and the relevant results were displayed in a Volcano plot (Figure 3a). The analyzed DEGs were then intersected with the genes highly expressed in Population 4 of NK cells, and 222 common genes were obtained (Figure 3b). GO enrichment analysis was then initiated on these common genes. The corresponding results have manifested that these genes were mainly enriched in DNA conformation change and nuclear division, chromosome segregation (Figure 3c). These findings thus hint at the potential modulatory effects of these overlapped genes on cell cycle and cell division so as to participate in the progression of HCC.

Figure 3Sorting on the feature genes and functional enrichment analysis. (a) Volcano plot on the DEGs from the HCC and normal samples based on the data from TCGA, with downregulated DEGs marked in blue, upregulated DEGs marked in red, and those without statistical significance marked in grey. (b) Venn diagram showing the common genes of the DEGs and the genes highly expressed in Population 4 of NK cells. (c) GO enrichment analysis result on the common genes from Venny analysis.

**Figure:**
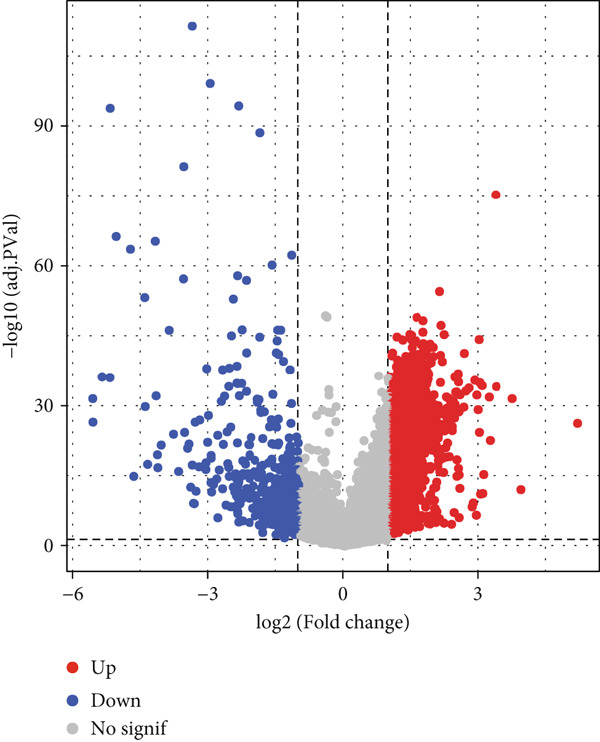
(a)

**Figure:**
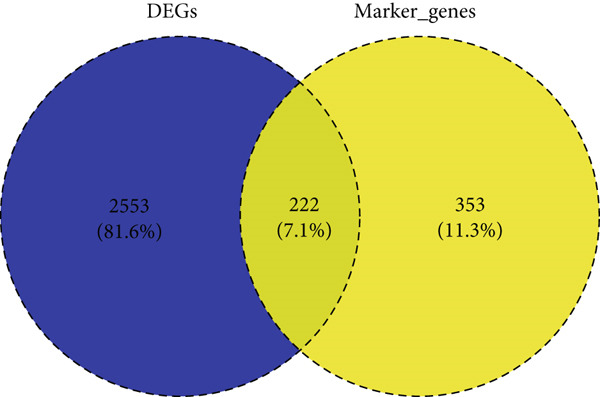
(b)

**Figure:**
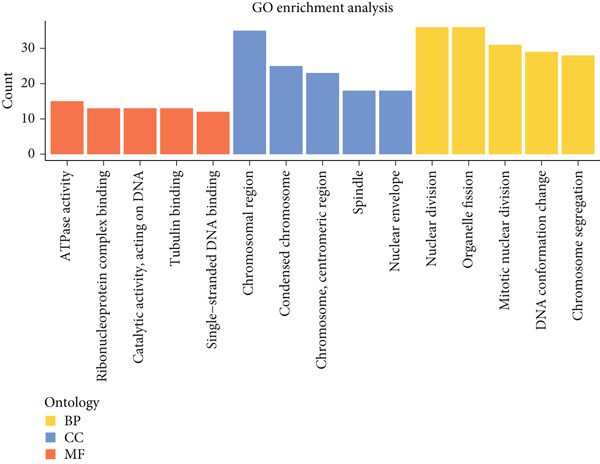
(c)

### 3.4. Construction and Validation on the Efficacy of the Risk Model

The univariate Cox regression analysis was then performed on the 222 common genes to sort the prognostically relevant genes. Further, LASSO regression analysis and stepwise regression were applied to reduce the number of these prognostically relevant genes. Finally, eight feature genes were identified and applied to construct the risk model using the formula below (Figures 4a,b):

Figure 4Construction and validation on the efficacy of the risk model. (a) Genes in the risk model and the correlation coefficient. (b) Multivariate forest plot of the genes in the risk model. (c–e) The overall survival in patients of high‐/low‐risk group using the data from TCGA‐LIHC (c), ICGC‐LIHC (d) and dataset GSE42619 (e). (f–h) The calculated AUC values based on the data from TCGA‐LIHC (f), ICGC‐LIHC (g) and dataset GSE42619 (h). ( ^∗^ *p* < 0.05,  ^∗∗^ *p* < 0.01, and  ^∗∗∗^ *p* < 0.001).

**Figure:**
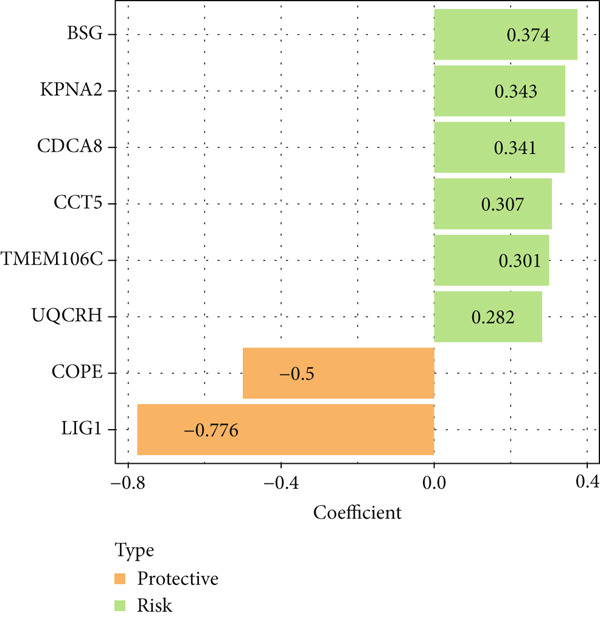
(a)

**Figure:**
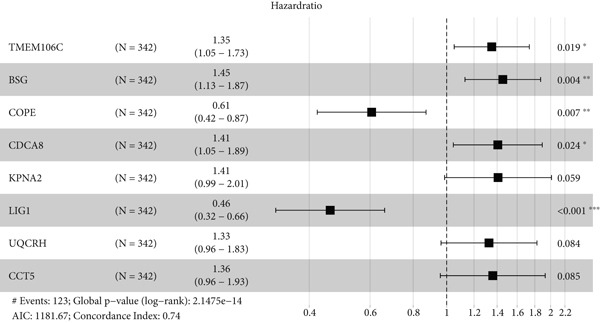
(b)

**Figure:**
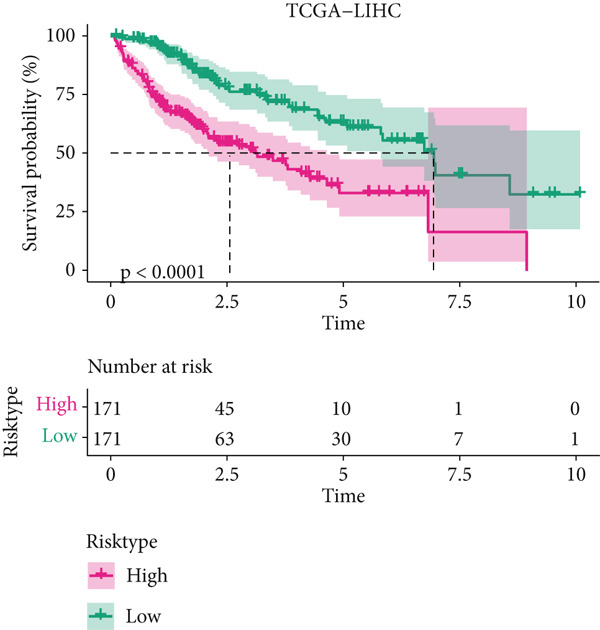
(c)

**Figure:**
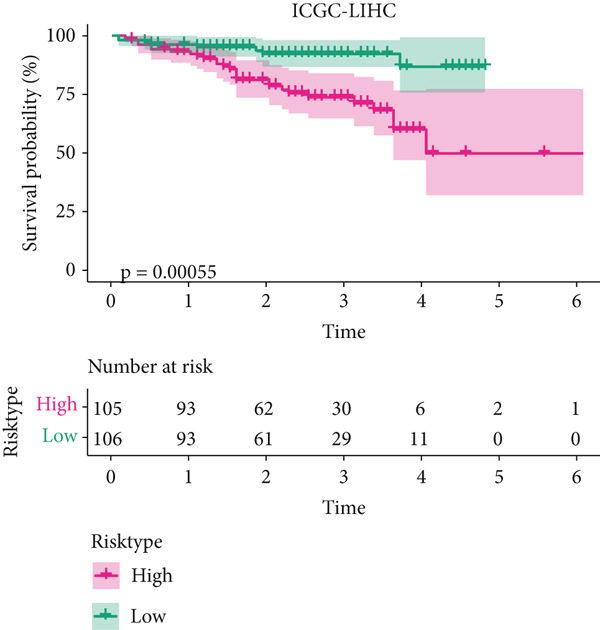
(d)

**Figure:**
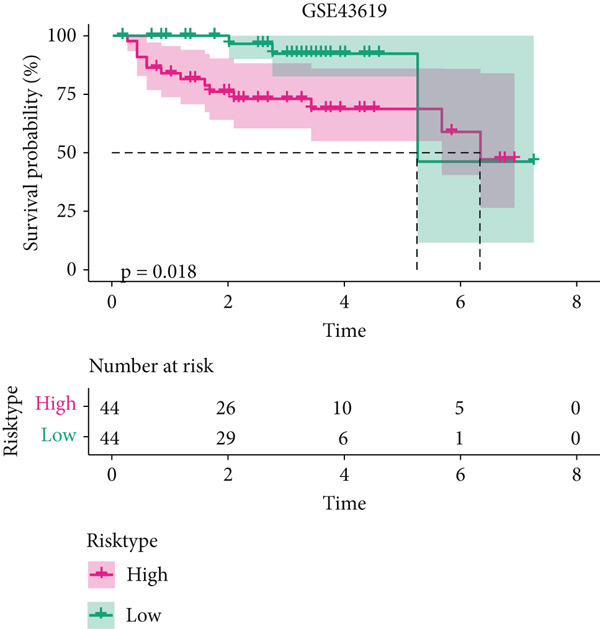
(e)

**Figure:**
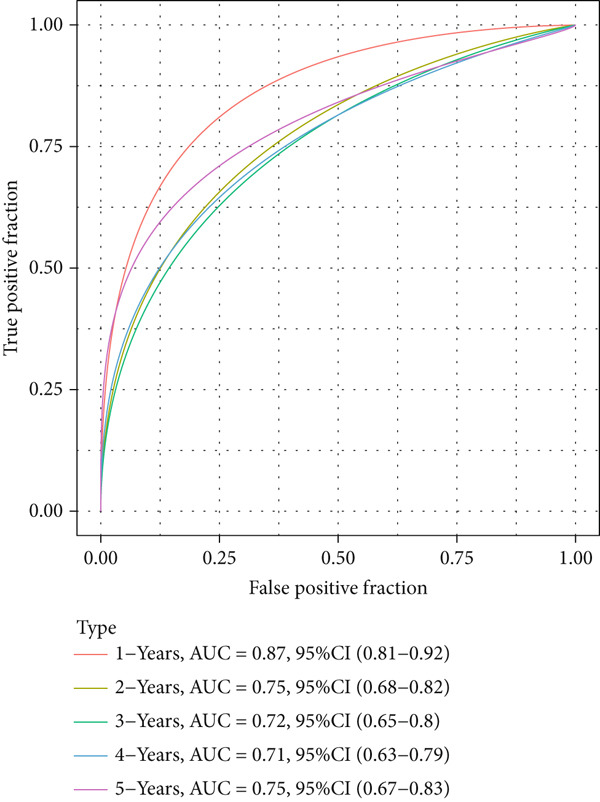
(f)

**Figure:**
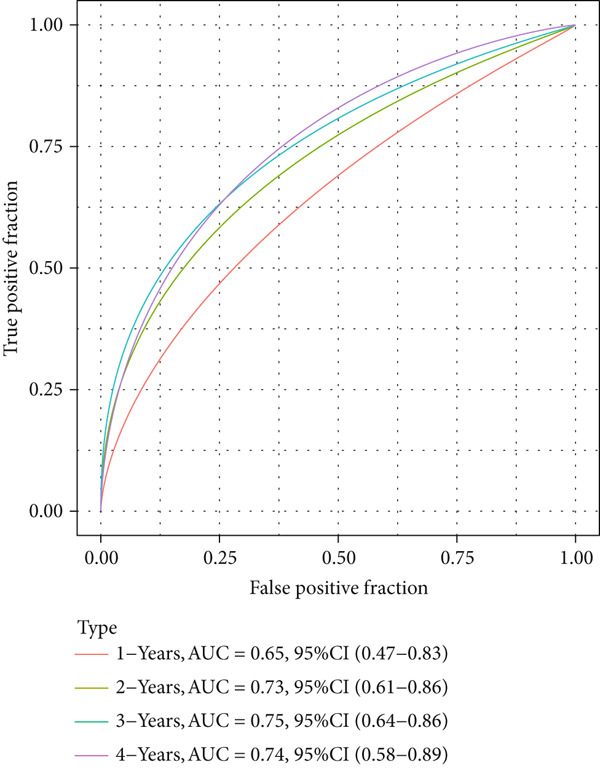
(g)

**Figure:**
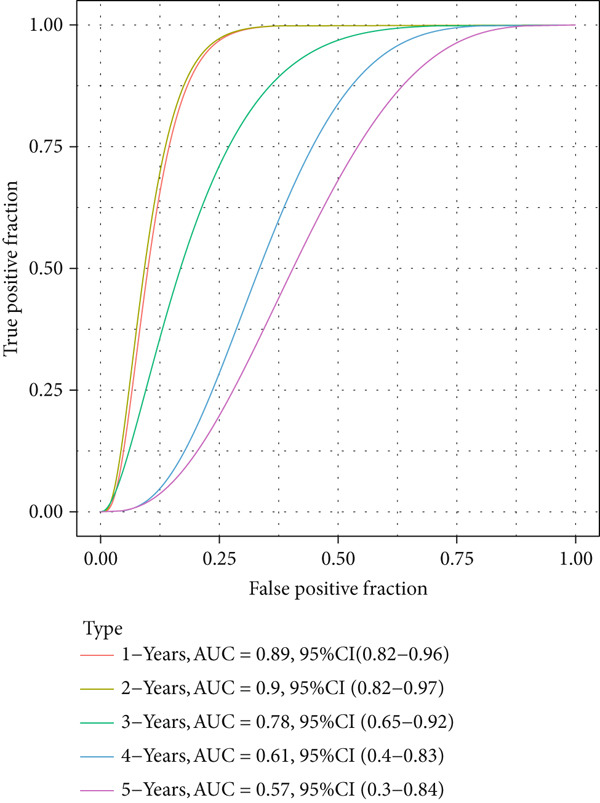
(h)

Risk score = 0.301∗*T* *M* *E* *M*106*C* + 0.374∗*B* *S* *G* + (−0.5∗*C* *O* *P* *E*) + 0.341∗*C* *D* *C* *A*8 + 0.343∗*K* *P* *N* *A*2 + (−0.776∗*L* *I* *G*1) + 0.282∗*U* *Q* *C* *R* *H* + 0.307∗*C* *C* *T*5

The risk score of each sample in TCGA cohort was calculated, and the samples were allocated based on the median of the calculated risk score to the high‐ or low‐risk group. The efficacy of the risk model was then validated in the cohorts of TCGA‐LIHC. Based on the results, the poorer prognosis was observed in patients of high‐risk group than that of low‐risk group (Figure 4c). Additional validation using ICGC‐LIHC and GSE43619 cohorts has shown that high‐risk group of patients tend to have a shorter overall survival (Figure 4d,e). The ROC curve and the AUC values were further plotted and calculated. In TCGA‐LIHC, the AUC value of the risk model in predicting the 1‐, 2‐, 3‐, 4‐, and 5‐year survival was 0.87, 0.75, 0.72, 0.71, and 0.75 (average: 0.76, Figure 4f). As to the ICGC‐LIHC‐JP cohorts, the AUC value of the risk model in predicting the 1‐, 2‐, 3‐, and 4‐year survival was 0.65, 0.73, 0.75, and 0.74 (average > 0.7, Figure 4g). Besides, in the dataset GSE43619, the AUC value of the risk model in evaluating the 1‐, 2‐, 3‐, 4‐, and 5‐year survival was 0.89, 0.9, 0.78, 0.61, and 0.57 (average: 0.75, Figure 4h). These discoveries hence demonstrated the efficacy of risk score in stratification of HCC patients into different risks.

Furthermore, a series of laboratory assays were carried out to validate the potential involvement of the eight feature genes in HCC. Based on the data from quantification test in HCC cells HuH7 and human immortal adult liver epithelial cell line THLE‐2, the differential expression of the eight feature genes in HCC cells was observed, with a profound elevated expression of *TMEM106C*, *CDCA8*, *KPNA2* and *UQCRH* in HCC cells (Figure S1a). Given that *CDCA8* is significantly overexpressed in HCC cells and has been clearly documented in the literature to promote mitotic chromosome segregation and HCC progression [26], it was selected for subsequent functional validation experiments. We validated the efficiency following *CDCA8* knockout (Figure S1b). Subsequently, according to the results of CCK‐8 (Figure S1c), scratch (Figure S1d), and Transwell assays (Figure S1e), the knockdown of *CDCA8* via small interfering RNA could repress the viability, migration, and invasion of HuH7 cells in vitro (Figures S1c, S1d, and S1e).

### 3.5. Nomogram for Precise Prognostication

To improve the prognostic assessment of risk score in clinical practice, both univariate and multivariate Cox regression analyses were carried out as needed. The results have manifested that both clinical stage and risk score were the independent prognosis factors (Figure 5a,b), hinting that these two factors may have a profound impact on the survival of HCC patients. Thereafter, a nomogram incorporating the clinical stage and risk score was established (Figure 5c). The corresponding calibration curve and decision curve analysis showed that the nomogram is close to the actual prediction on the 1‐, 3‐ and 5‐year survival (Figure 5d) and that the integration of clinical stage and risk score can provide more support on clinical decision than the application of clinical stage or other prognostic model alone (Figure 5e), thus demonstrating the potential of nomogram on precise prognostication in HCC.

Figure 5Nomogram for precise prognostication. (a–b) Univariate and multivariate Cox regression analysis on the risk score and clinical stage. (c) Nomogram incorporating risk score and clinical stage. (d–e) Calibration curve and decision curve of the nomogram. The data with statistical significance were denoted with asterisks ( ^∗∗∗^ *p* < 0.001).

**Figure:**
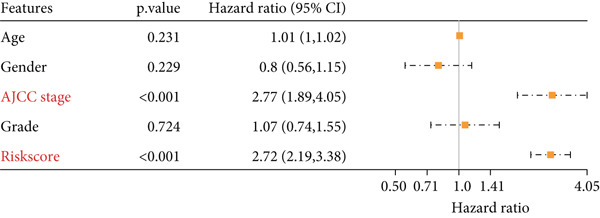
(a)

**Figure:**
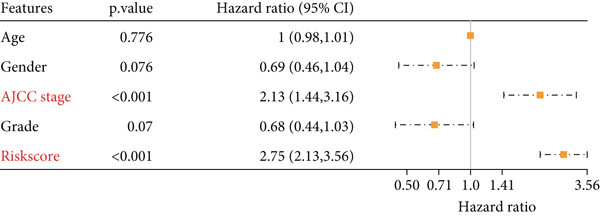
(b)

**Figure:**
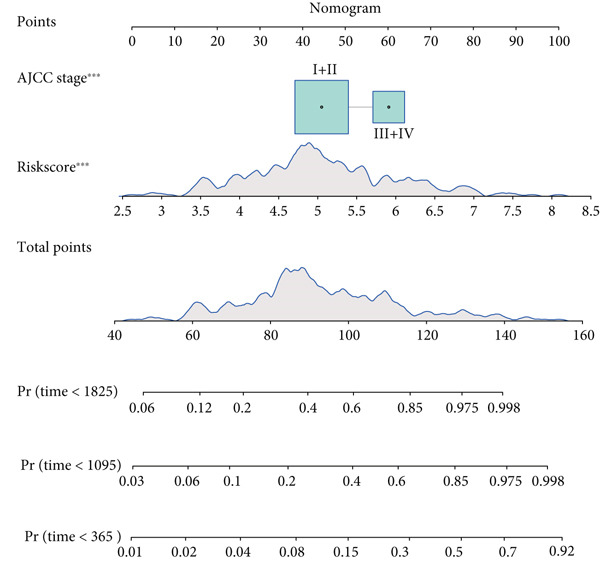
(c)

**Figure:**
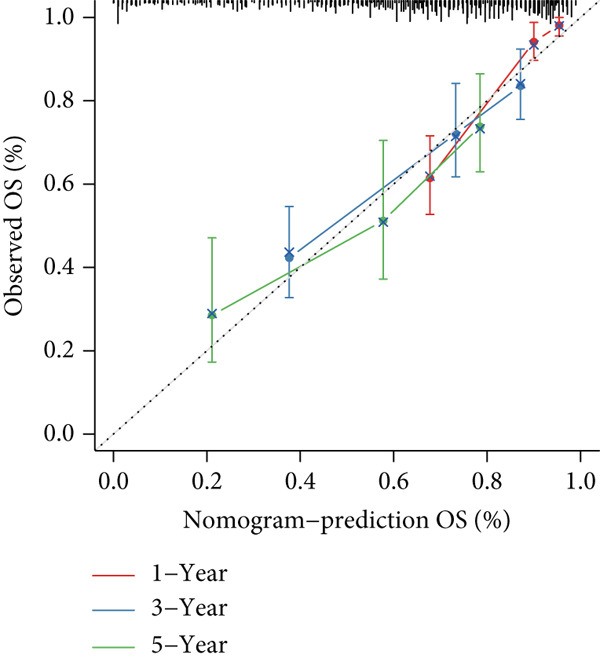
(d)

**Figure:**
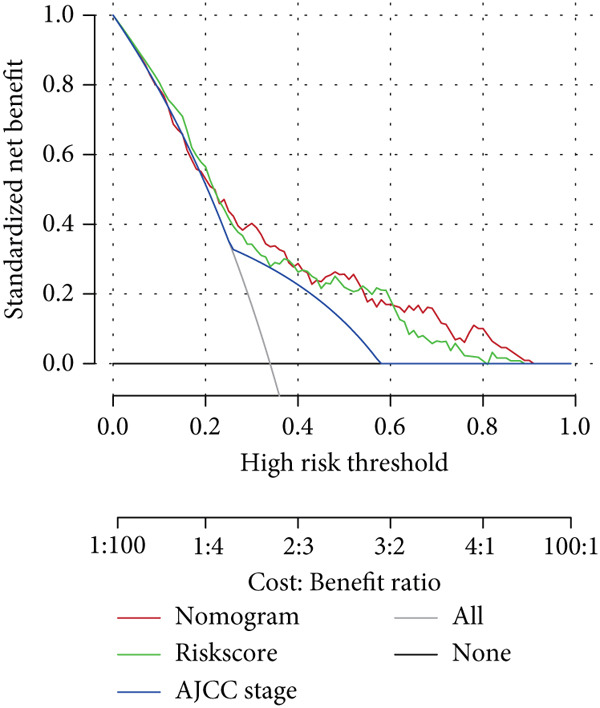
(e)

### 3.6. Landscape of Immune Infiltration

The immune infiltration status of TCGA‐LIHC data was analyzed in the following three algorithms to reveal the potential effects of tumor microenvironment (TME) on the prognostication of HCC. Firstly, the immune infiltration score of each sample in TCGA‐LIHC was determined using ssGSEA, revealing that the score was evidently higher in the samples of high risk. In particular, the infiltration degree of myeloid‐derived suppressor cells (MDSC), T cells (regulatory T cells, activated CD4^+^ T cells, central memory CD8^+^ T cells), and dendritic cells (activated dendritic cells and immature dendritic cells) was evidently higher in the samples of high risk (Figure 6a).

Figure 6Landscape of immune infiltration. (a) The infiltration score of tumor‐infiltrating lymphocytes in TCGA‐LIHC with high/low risk score. (b–c) Difference in the TIMER score and MCPCounter score of the tumor‐infiltrating lymphocytes in TCGA‐LIHC with high/low risk score. The data with statistical significance were denoted with asterisks ( ^∗^ *p* < 0.05,  ^∗∗^ *p* < 0.01,  ^∗∗∗^ *p* < 0.001, and  ^∗∗∗∗^ *p* < 0.0001) and those without statistical significance were denoted with “ns” (*p* > 0.05).

**Figure:**
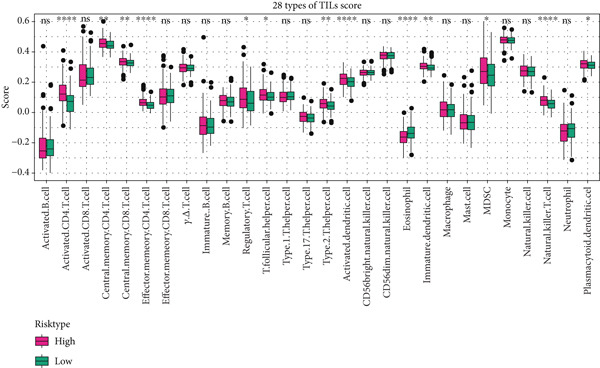
(a)

**Figure:**
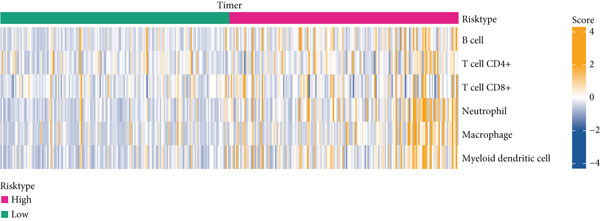
(b)

**Figure:**
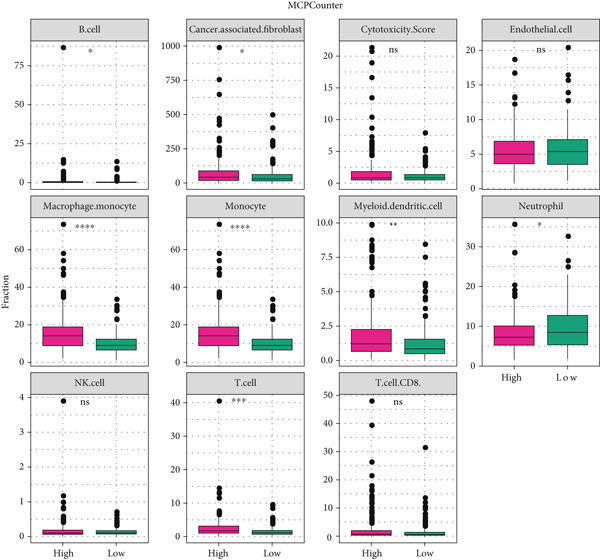
(c)

Secondly, the infiltration status of TILs was evaluated using the TIMER algorithm, and the corresponding results have manifested that the score of TILs in the samples of high risk (Figure 6b). Additionally, MCPCounter analysis suggested the high infiltration of TILs in the samples of high risk (Figure 6c). These evidences collectively demonstrated the high immune infiltration in HCC patients of high risk.

### 3.7. Predictive Value of Risk Score on the Immunotherapy Response in HCC Patients

The TIDE algorithm was employed to evaluate the therapeutic response of HCC patients to the immune checkpoint inhibitors (ICIs) using the data from TCGA‐LIHC. It was illustrated that the percentage of patients at high risk to ICIs was evidently lower than that of low risk (Figure 7a), suggesting the poor response to immunotherapy. Further analyses have unveiled that the TIDE score and the immune evasion‐related score (exclusion score) were higher in HCC patients at high risk (Figure 7b,c). Such discoveries hinted at the possible involvement of immune evasion in these patients at high risk. Additionally, comparison of the expression levels of immune checkpoint–relevant genes showed higher expression levels of these genes in HCC patients at high risk (Figure 7d).

Figure 7Predictive value of risk score on the immunotherapy response in HCC patients. (a) Percentage of patients responding to therapies using immune checkpoint inhibitors. (b–c) The calculated TIDE score and exclusion score in patients of high‐/low‐risk group. (d) The heatmap showing the expression levels of immune checkpoint‐related genes in patients of high‐/low‐risk group. The data with statistical significance were denoted with asterisks ( ^∗∗∗∗^ *p* < 0.0001).

**Figure:**
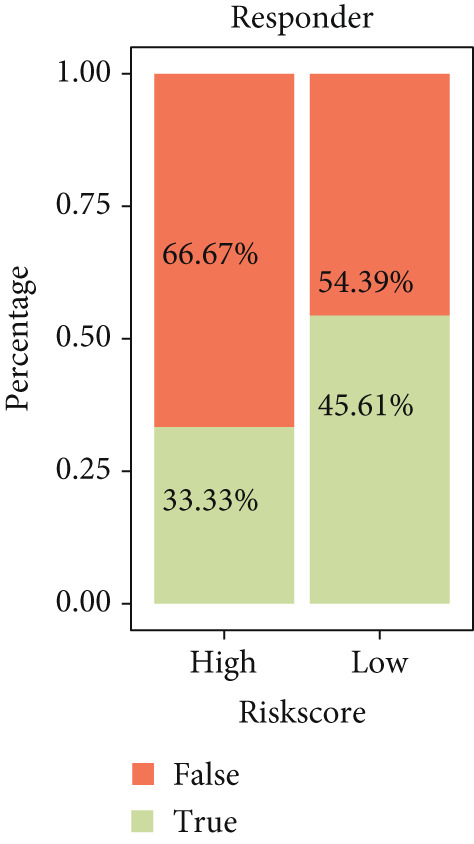
(a)

**Figure:**
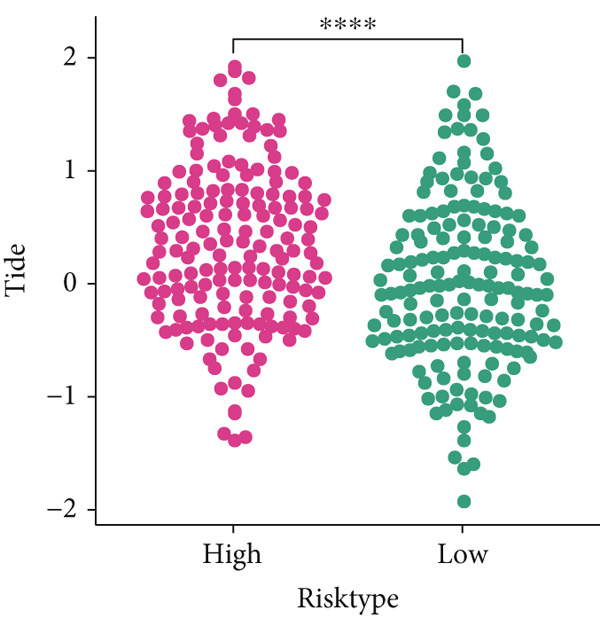
(b)

**Figure:**
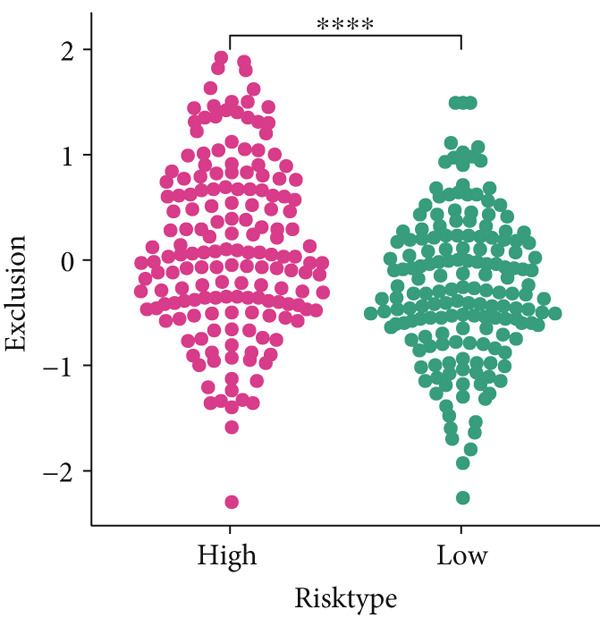
(c)

**Figure:**
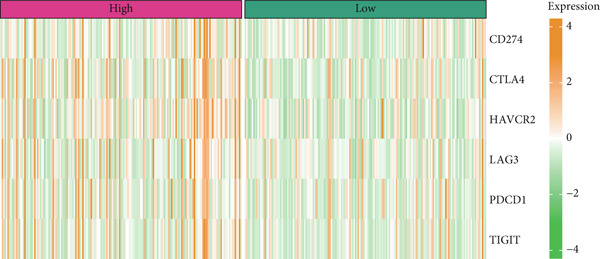
(d)

## 4. Discussion

HCC continues to pose a significant clinical challenge due to its high recurrence rate and suboptimal response to current therapies, particularly immunotherapies [28]. Although cellular senescence has been implicated in tumor biology, its specific role in shaping the HCC immune microenvironment and its translational potential as a predictive biomarker remain poorly defined, underscoring the urgent need for integrative analyses. To address this gap, our study innovatively combined single‐cell and bulk transcriptomic data to identify a high‐senescence NK cell subpopulation and subsequently constructed an eight‐gene senescence‐related signature. This signature not only robustly stratifies HCC patients into distinct prognostic groups but also, for the first time, reveals a critical link between a senescence‐high phenotype and a dysfunctional immune microenvironment characterized by high infiltration yet poor predicted response to immune checkpoint inhibitors. Collectively, our work provides a novel, clinically applicable tool for risk assessment and highlights cellular senescence as a key determinant of immunotherapy resistance, offering a promising avenue for developing combination strategies to improve outcomes in HCC patients.

Accurate prediction for patients′ survival outcomes and treatment responsiveness is fundamental for advancing personalized treatment in precision oncology [29, 30]. With the help of scRNA‐seq, a variety of cell types are implicated in the progression of HCC, like cancer‐associated fibroblasts [31], tumor‐associated neutrophils [32], and cancer stem cells [33], to name a few. Cellular senescence in HCC has been underscored to play a crucial role and regarded as a fail–safe program which can inhibit cell growth and promote tissue repair or the progression of chronic inflammatory liver diseases to trigger carcinogenesis [34, 35]. While linking scRNA‐seq with senescence in the field of oncology, a senescence‐based gene signature has been proposed in the research of colorectal cancer, which also identified the role of SPP1‐positive macrophages in the senescence of colorectal cancer [36]. In our current study, with the purpose of exploring some senescence‐related biomarkers in HCC, scRNA‐seq analysis was firstly applied to identify the cell cluster for our research. Based on the dataset GSE162616, NK cells were recognized as the cell cluster with a relatively higher percentage in HCC sample. It has been documented that NK cells take up 25%–50% of lymphocytes in the liver and that the number of NK cells in the tumor tissues and blood derived from patients with HCC is positively linked to the prognosis and patients′ survival, thus demonstrating the role of NK cells in liver immunity [37]. These evidences collectively demonstrated the critical role of senescence in HCC development.

Thereafter, we are aimed at figuring out the potential biomarkers for our research. Toward this end, the genes highly expressed in Population 4 of NK cells (the population of NK cells with the highest senescence score) were intersected with the DEGs in both HCC and normal tissue based on the TCGA data. The subsequent enrichment analysis has revealed the enrichment of these common genes in chromosome segregation, DNA conformation change and nuclear division. Chromosome segregation refers to the partitioning of genetic material into two daughter cells and is one of the most critical processes in cell division [38]. A lesion in the chromosome segregation may lead to the occurrence of chromosome instability, a property which is related to diverse cancer cells and contributes to aging [39, 40]. DNA conformation change is also a crucial mechanism for gene regulation during the development and disease like senescence [41, 42]. Besides, the altered architecture of cell nuclei is often observed in malignant cells and is associated with the cancers, which provides a crucial diagnostic feature [43]. These evidences thus hinted at the possible modulation of these overlapped genes on cell cycle to control the progression of HCC. Then, to narrow down the number of genes of interest for our research, the prognostically relevant genes were additionally identified from the overlapped genes, and eight key genes were accordingly identified. *TMEM106C* belongs to the transmembrane protein family which contributes to the malignant phenotypes and poor prognosis of HCC [44]. *BSG* is alternatively known as cluster of differentiation 147 and plays a fundamental role in the intercellular recognition implicated in immunologic phenomena, differentiation and development, which can also predict the prognosis in HCC [45, 46]. *COPE* belongs to the COPI coatomer complex with effects in HCC awaiting to be elucidated in detail [47]. *CDCA8* is essential for chromosomal segregation during mitosis, which acts as an oncogene promoting the progression of HCC [26]. *KPNA2* is a member of the karyopherin *α*/importin *α* family, which involves in the classical nuclear protein import pathway and promotes the progression of HCC [48]. *LIG1* is also shown to express highly in cancer cell lines and its involvement in HCC requires additional validation [49]. *UQCRH* is a mitochondrial hinge protein promoting the proliferation, migration and energy metabolism of HCC [50, 51]. Besides, *CCT5* belongs to the chaperonin‐containing TCP1 complex which is upregulated in liver tumors and can predict the shortened overall survival and disease‐free survival [52]. These eight genes were incorporated to construct a risk score model which has a strong prognostication efficacy.

The patients of HCC were allocated to high or low risk score by the median value and the immune infiltration status was determined. The TME has been documented to be composed of various cell types, including mesenchymal cells and resident and infiltrated immune cells [53]. Prediction of clinical outcome and development of relevant immunotherapies involve systematic interrogation of tumor‐infiltrating immune cells [54]. As the major component cells of TILs, T cells are proved to exert either antitumor or tumor‐promoting effects on HCC [55]. Relevant results of this study have manifested the higher percentage of immune cells in HCC patients of high risk score. Specifically, the percentage of T cells (central memory CD8^+^ T cells, activated CD4^+^ T cells, and regulatory T cells), dendritic cells (activated dendritic cells and immature dendritic cells), and MDSCs were higher in HCC patients of high risk score. Similar results were also noticed in some existing researches addressing the TILs in HCC [56–60]. These findings collectively underscore that a high‐risk senescence‐related gene signature is associated with an immunosuppressive tumor microenvironment characterized by abundant yet dysfunctional immune infiltration, which may underlie the diminished response to immunotherapy observed in these patients.

This study has several limitations that should be acknowledged. First, the prognostic model was developed and validated using retrospective data from public repositories. To establish its clinical utility, future work should involve prospective validation in a multicenter cohort, with standardized sample collection and long‐term follow‐up to assess the model′s performance in real‐world clinical settings. Second, our analysis is primarily based on transcriptomic data. Protein‐level validation of the key signature genes (e.g., via immunohistochemistry on tissue microarrays or Western blot) is necessary to confirm their expression and biological relevance in HCC tissues. Integrating proteomic data in future studies would provide a more complete functional picture. Third, the cohorts used predominantly represent certain geographic and ethnic populations. Extending validation to more diverse, independent cohorts—particularly from regions with different etiological backgrounds—is essential to evaluate the generalizability of the senescence signature. Finally, although in vitro experiments supported the role of *CDCA8*, the lack of in vivo functional validation limits the mechanistic understanding of how these genes influence the tumor immune microenvironment. Future studies should employ genetically engineered mouse models or patient‐derived xenografts with modulation of key genes (e.g., *CDCA8* knockdown/overexpression) to systematically investigate their impact on tumor growth, senescence induction, and response to immunotherapy in a physiological context. Addressing these points will strengthen the translational potential of our findings and provide deeper insights into senescence‐mediated immunomodulation in HCC.

## 5. Conclusion

This study constructed an eight‐gene senescence‐related signature (*TMEM106C*, *BSG*, *COPE*, *CDCA8*, *KPNA2*, *LIG1*, *UQCRH*, and *CCT5*) from a high‐senescence NK cell subpopulation in HCC. The validated model stratifies patients into distinct prognostic groups and uniquely predicts poorer response to immune checkpoint inhibitors in high‐risk patients, linking cellular senescence to an immunosuppressive microenvironment. Functional assays confirmed the oncogenic role of *CDCA8*. These findings provide a practical tool for risk assessment and highlight senescence modulation as a potential strategy to overcome immunotherapy resistance in HCC.

**Nomenclature**
HCC: hepatocellular carcinoma
SASP: senescence‐associated secretory phenotype
SRGs: senescence‐related genes
TCGA: the Cancer Genome Atlas
RNA‐seq: RNA sequencing
scRNA‐seq: single‐cell RNA sequencing
LIHC: liver hepatocellular carcinoma
PCA: principal component analysis
DEGs: differentially expressed genes
GO: gene ontology
BP: biological process
MF: molecular function
CC: cellular component
ROC: receiver operator characteristic
AUC: area under the curve
TILs: tumor‐infiltrating lymphocytes
FBS: fetal bovine serum
NK: natural killer
TME: tumor microenvironment
MDSC: myeloid‐derived suppressor cells
ICIs: immune checkpoint inhibitors

## Ethics Statement

Ethical approval was not required for this study because it is not involved in any human experiments.

## Consent

The authors have nothing to report.

## Disclosure

All authors read and approved the manuscript.

## Conflicts of Interest

The authors declare no conflicts of interests.

## Author Contributions

All authors contributed to this present work: K.Y., J.D., and E.H. designed the study; J.L., Q.L., and W.Z. acquired the data; K.Y. and M.C. interpreted the data. K.Y. and W.H. drafted the manuscript, Z.D. and X.H. revised the manuscript.

## Funding

This study was supported by the Natural Science Foundation of Guangxi Zhuang Autonomous Region (10.13039/100012547; 2025GXNSFBA069220) and the Talent Education Program of Guangxi Science and Technology Project (No: Guike AA23026008).

## Supporting Information

Additional supporting information can be found online in the Supporting Information section.

## Supporting information


**Supporting Information 1** Figure S1. Laboratory validation using HCC cells HuH7 to test the involvement of the feature genes in HCC. (a) The quantified mRNA levels of the eight feature genes (for the risk score model) in HCC cells HuH7 and human immortal adult liver epithelial cell line THLE‐2. (b) The knockdown efficiency of *CDCA8*‐specific small interfering RNAs in HCC cells HuH7. (c) The quantified OD_450_ value in HCC cells HuH7 with or without the silencing of *CDCA8* at 0, 24, 48 and 72 h based on the results of CCK‐8 assay. (d–e) Scratch and Transwell assay on evaluating the in vitro migration and invasion of HCC cells HuH7 with or without the silencing of *CDCA8*. The data with statistical significance were denoted with asterisks ( ^∗∗∗^ *p* < 0.001, vs. THLE‐2;  ^∧∧^ *p* < 0.01,  ^∧∧∧^ *p* < 0.001, and  ^∧∧∧∧^ *p* < 0.0001) and those without statistical significance were denoted with “ns” (*p* > 0.05).

**Supporting Information 2** Table S1. The target sequence for transfection via liposome.

**Supporting Information 3** Table S2. Forward and reverse primers for PCR assay.

## Data Availability

The datasets generated and/or analyzed during the current study are available in the GSE162616 repository (https://www.ncbi.nlm.nih.gov/geo/query/acc.cgi?acc=GSE162616), GSE42619 repository (https://www.ncbi.nlm.nih.gov/geo/query/acc.cgi?acc=GSE42619), and GSE43619 repository (https://www.ncbi.nlm.nih.gov/geo/query/acc.cgi?acc=GSE43619).
