# Supplementary figures and images for: TMEM106C, BSG, COPE, CDCA8, KPNA2, LIG1, UQCRH, and CCT5: Predictive of Survival and Immunotherapy Resistance in Hepatocellular Carcinoma
Source: Hum Mutat. 2026 Feb 10;2026:1465989. doi: 10.1155/humu/1465989 (PMC12887829; doi:10.1155/humu/1465989)

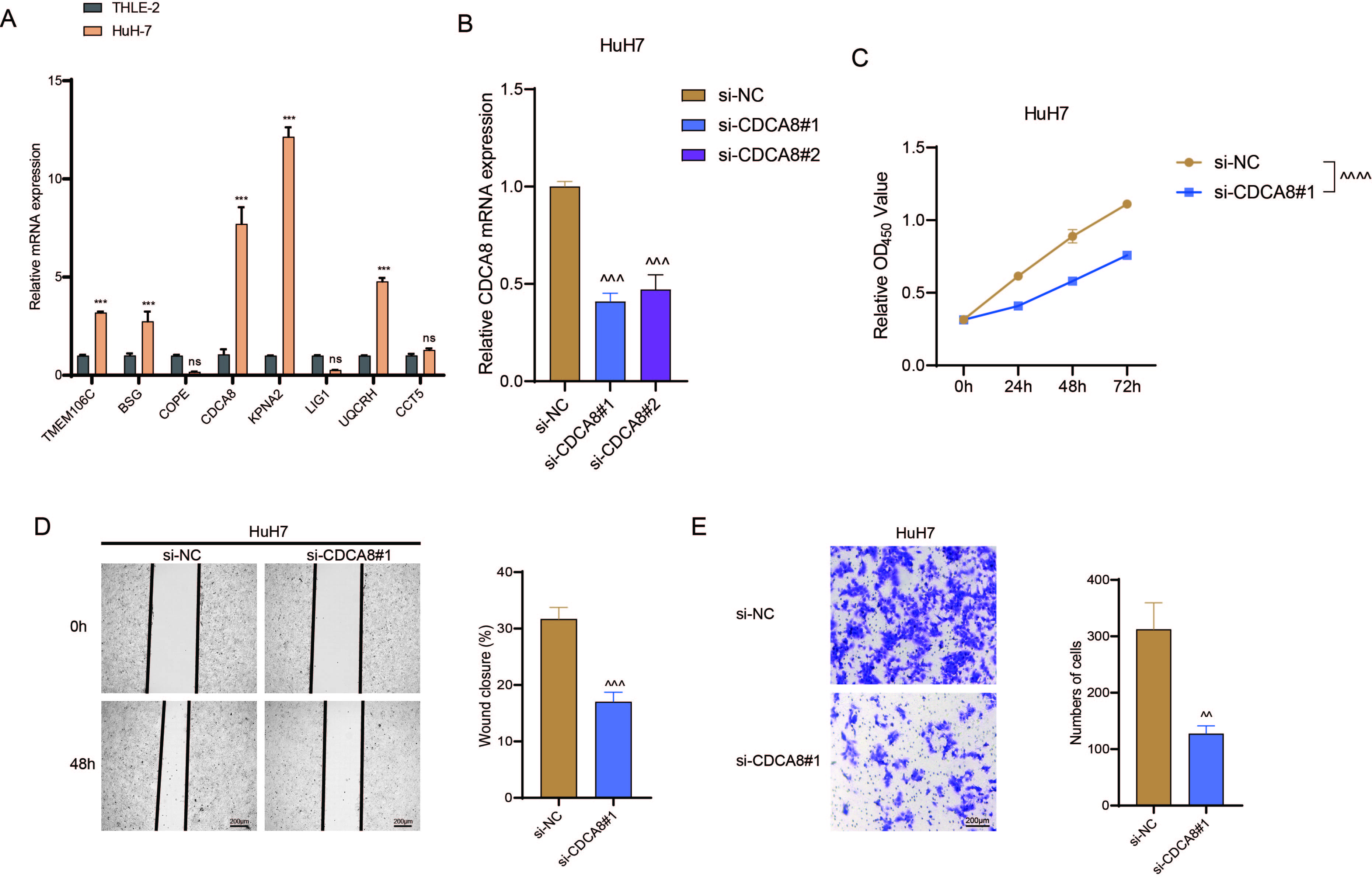

Supplement: Supplementary file 1 — Supporting Information 1 Figure S1. Laboratory validation using HCC cells HuH7 to test the involvement of the feature genes in HCC. (a) The quantified mRNA levels of the eight feature genes (for the risk score model) in HCC cells HuH7 and human immortal adult liver epithelial cell line THLE‐2. (b) The knockdown efficiency of CDCA8‐specific small interfering RNAs in HCC cells HuH7. (c) The quantified OD450 value in HCC cells HuH7 with or without the silencing of CDCA8 at 0, 24, 48 and 72 h based on the results of CCK‐8 assay. (d–e) Scratch and Transwell assay on evaluating the in vitro migration and invasion of HCC cells HuH7 with or without the silencing of CDCA8. The data with statistical significance were denoted with asterisks ( ∗∗∗ p < 0.001, vs. THLE‐2; ∧∧ p < 0.01, ∧∧∧ p < 0.001, and ∧∧∧∧ p < 0.0001) and those without statistical significance were denoted with “ns” (p > 0.05). [file HUMU-2026-1465989-s001.jpg]
