# Supplementary material for: TMEM106C, BSG, COPE, CDCA8, KPNA2, LIG1, UQCRH, and CCT5: Predictive of Survival and Immunotherapy Resistance in Hepatocellular Carcinoma
Source: Hum Mutat. 2026 Feb 10;2026:1465989. doi: 10.1155/humu/1465989 (PMC12887829; doi:10.1155/humu/1465989)
Supplement: Supplementary file 2 — Supporting Information 2 Table S1. The target sequence for transfection via liposome. [file HUMU-2026-1465989-s003.docx]

Table S1. The target sequence for transfection via liposome

| Gene | Target sequence (5’-3’) |
| --- | --- |
| si-CDCA8#1 | TACGAATCAAGCAAATTGAGTCA |
| si-CDCA8#2 | AGGTGGATAACCTCTACAACATC |
| si-NC | TCGTAGCATCTAACCGGATACAA |
