# Supplementary material for: TMEM106C, BSG, COPE, CDCA8, KPNA2, LIG1, UQCRH, and CCT5: Predictive of Survival and Immunotherapy Resistance in Hepatocellular Carcinoma
Source: Hum Mutat. 2026 Feb 10;2026:1465989. doi: 10.1155/humu/1465989 (PMC12887829; doi:10.1155/humu/1465989)
Supplement: Supplementary file 3 — Supporting Information 3 Table S2. Forward and reverse primers for PCR assay. [file HUMU-2026-1465989-s002.docx]

Table S2. Forward and Reverse Primers for PCR assay

| Gene | Primers (5’-3’) |
| --- | --- |
| *TMEM106C* forward | ATCAGGAACTCCAACTTCTA |
| *TMEM106C* reverse | GCATGAAGATCACTATGTTG |
| *BSG* forward | TTCACTACCGTAGAAGACCT |
| *BSG* reverse | TTCTCAATGTGTAGCTCTGA |
| *COPE* forward | CTTCCTGTATAGAGCGTACC |
| *COPE* reverse | CTCCTGGAAGATGTAGTAGG |
| *CDCA8* forward | ATGAACTGGCTTGACTACTT |
| *CDCA8* reverse | ACCTGTATTACCTTTCGTGT |
| *KPNA2* forward | GTCACTGGTACAGATGAACA |
| *KPNA2* reverse | GTTCCACCACTGGTATAGTT |
| *LIG1* forward | AGAGGGTAAAGCAAAGAAG |
| *LIG1* reverse | ACGGGATGATAGTTGTTCT |
| *UQCRH* forward | CGAGCAAAAGATGCTTAC |
| *UQCRH* reverse | AGAGGATACACGCTCATC |
| *CCT5* forward | CGTCTTATGGGACTTGAG |
| *CCT5* reverse | CTAGGTTAGCACCAGTCTCT |
| *GAPDH* forward | ATTGACCTCAACTACATGGT |
| *GAPDH* reverse | CATACTTCTCATGGTTCACA |
